# Robotic fabrication of high-quality lamellae for aberration-corrected transmission electron microscopy

**DOI:** 10.1038/s41598-021-00595-x

**Published:** 2021-11-03

**Authors:** Hideyo Tsurusawa, Nobuto Nakanishi, Kayoko Kawano, Yiqiang Chen, Mikhail Dutka, Brandon Van Leer, Teruyasu Mizoguchi

**Affiliations:** 1 Thermo Fisher Scientific, FEI Japan Ltd., 4-12-2, Higashi-Shinagawa, Shinagawa-ku, Tokyo, 140-0002 Japan; 2 Thermo Fisher Scientific, Achtseweg Noord 5, 5651 GG Eindhoven, The Netherlands; 3 Thermo Fisher Scientific, 5350 NE Dawson Creek Drive, Hillsboro, OR 97124 USA; 4 Institute of Industrial Science, University of Tokyo, 4-6-1 Komaba, Meguro-ku, Tokyo, 153-8505 Japan

**Keywords:** Transmission electron microscopy, Transmission electron microscopy, Characterization and analytical techniques

## Abstract

Aberration-corrected scanning transmission electron microscopy (STEM) is widely used for atomic-level imaging of materials but severely requires damage-free and thin samples (lamellae). So far, the preparation of the high-quality lamella from a bulk largely depends on manual processes by a skilled operator. This limits the throughput and repeatability of aberration-corrected STEM experiments. Here, inspired by the recent successes of “robot scientists”, we demonstrate robotic fabrication of high-quality lamellae by focused-ion-beam (FIB) with automation software. First, we show that the robotic FIB can prepare lamellae with a high success rate, where the FIB system automatically controls rough-milling, lift-out, and final-thinning processes. Then, we systematically optimized the FIB parameters of the final-thinning process for single crystal Si. The optimized Si lamellae were evaluated by aberration-corrected STEM, showing atomic-level images with 55 pm resolution and quantitative repeatability of the spatial resolution and lamella thickness. We also demonstrate robotic fabrication of high-quality lamellae of SrTiO_3_ and sapphire, suggesting that the robotic FIB system may be applicable for a wide range of materials. The throughput of the robotic fabrication was typically an hour per lamella. Our robotic FIB will pave the way for the operator-free, high-throughput, and repeatable fabrication of the high-quality lamellae for aberration-corrected STEM.

## Introduction

Aberration-corrected scanning transmission electron microscopy (STEM) is widely used for atomic-level imaging of materials^1,2^, offering spatial resolution in a sub-50-pm range^3,4^ and powerful micro-analysis technology^5–8^. Since atomic-level microstructures often link with material properties, aberration-corrected STEM imaging plays a central role in materials discovery^9,10^. However, a fundamental problem remains in the preparation of STEM samples. Aberration-corrected STEM usually requires a thin sample (lamella) with thickness below 50 nm and a damage-free surface. Current methods to prepare such high-quality lamellae from bulk materials largely depend on manual processes and the knowledge of a skilled user. These not only limit the throughput of the full workflow of aberration-corrected STEM experiments but also suppress the repeatability of the sample quality and the resulting STEM images. We note that the throughput and repeatability have a critical impact when aberration-corrected STEM analysis is used in a study of high-throughput experiments, such as combinatorial chemistry^11^.

**Figure 1:**
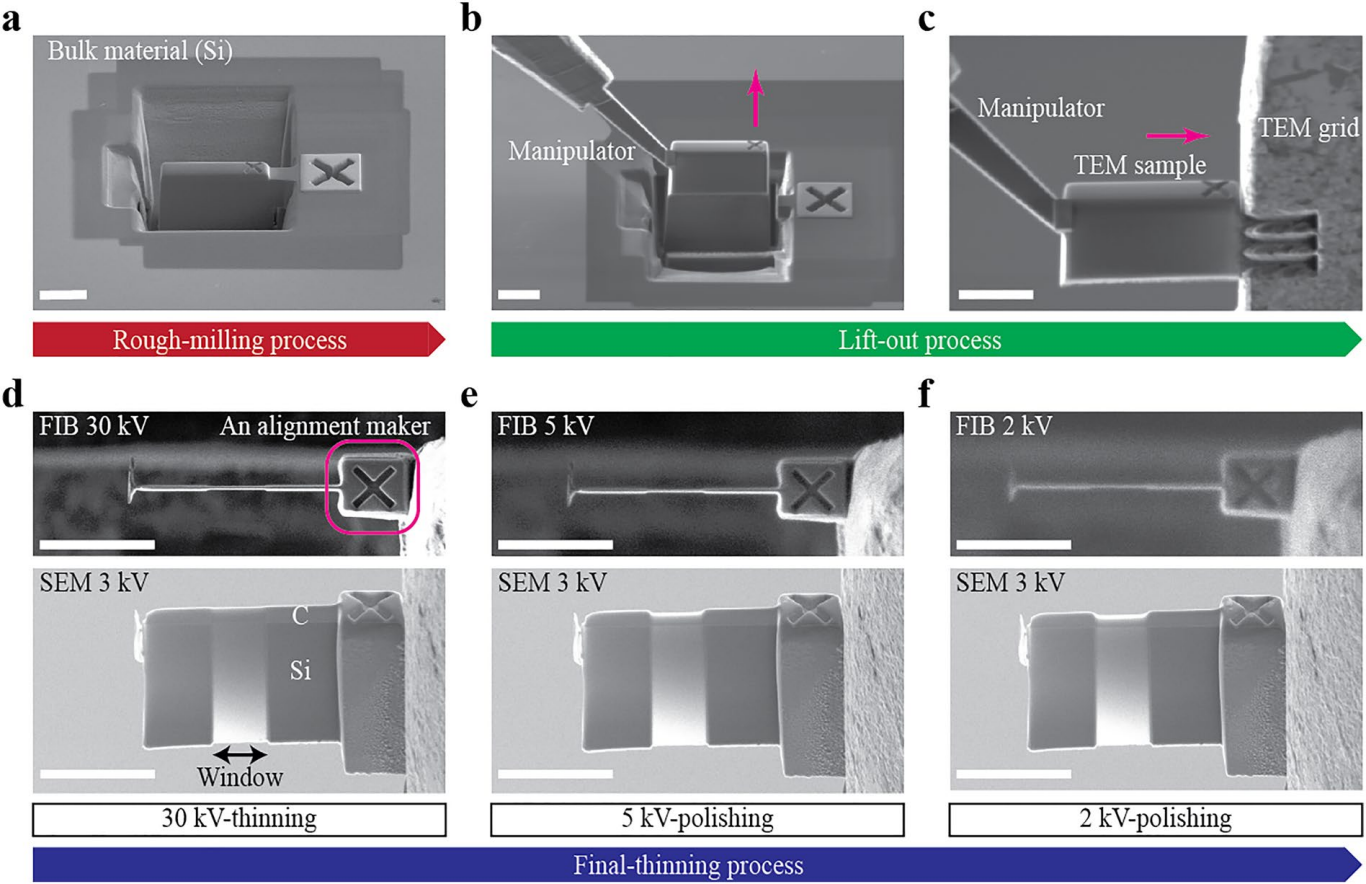
Robotic FIB operation to prepare a high-quality lamella. (**a**) Rough-milling process to prepare a chunk in a bulk. (**b**) The in-situ manipulator lifts out the chunk. (**c**) The chunk is transferred and fixed to a TEM grid at the position. After the FIB then cuts the tip of the manipulator to release the sample, the final-thinning process starts. (**d**) FIB and SEM (secondary electron) images after 30 kV thinning. Finally, the FIB thins and polishes a narrower “window” region of approximately 2.4 μm wide. (**e,f**) FIB and SEM images after FIB polishing at 5 kV and 2 kV, respectively. Preparing one STEM sample takes about 1 h (see Table 1). The scale bars shown in both FIB and SEM images are 5 μm long.

Focused-ion-beam (FIB) is one of the common methods to prepare high-quality lamellae for aberration-corrected STEM imaging^12–14^. In a standard experiment, such a lamella is prepared by three main steps: rough-milling, lift-out, and final-thinning (see also Fig. 1). In the rough-milling process, FIB is used to extract a thin chunk from the bulk material. Then, the chunk is transferred to a TEM grid using an in-situ manipulator (the lift-out process). In the final-thinning process, FIB thins and polishes the chunk down to the order of 10 nm in thickness. A number of studies on functional materials have used FIB for preparing lamellae that yield high-quality atomic-level images^15–22^. So, one may suggest the automation of the FIB operation as an ideal solution to achieve the high throughput and repeatability of preparing high-quality lamellae.

The concept of such science automation (or robot scientists) has been widely demonstrated in biology^23,24^, chemistry^25,26^, and materials science^27–29^, where a robotically-controlled system conducts a set of templated experiments without the attendance of a human operator. In general, the robot scientists realize a higher throughput than a conventional method by manual operations. Moreover, the digitalized control by a robot scientist often offers better precision and repeatability. So, we aim to establish an operator-free, high-throughput, and repeatable method to fabricate high-quality STEM lamellae by a robotic FIB system.

A primary challenge of the robotic FIB is the automatic control of the final-thinning process and the optimization of the FIB parameters. Aberration-corrected STEM usually requires that the lamella should have a thickness below 50 nm and still maintain intact (damage-free) surfaces. To improve the surface quality, the FIB acceleration voltage should be reduced from 30 to 2 kV (or below) in the final-thinning process^12–14^. However, low-kV FIB images are typically blurred and of poor quality. Even when a skilled user manually operates the FIB, the low-kV polishing process usually faces two complex problems. (1) Where should the FIB milling box be set in the blurred FIB image? (2) When should the final-polishing process be terminated? In a conventional procedure of manual FIB operation, the operator sets the FIB milling box in the FIB image by eye (i.e., without any digitalized correction of the milling position). During the FIB thinning/polishing, the operator carefully monitors the lamella shape and its thickness (transparency) via SEM imaging to determine the end-point of each FIB process. Hence, the final-thinning process of a manual FIB heavily depends on the skill and experience of the operator. These operator-dependent processes primarily limit the repeatability and its throughput of preparing high-quality lamellae.

We consider a simplified strategy of the final-thinning process to fit a robotic way. First, we assume that automatic image recognition can set the milling box in the FIB image with much higher precision than a human operator does. This may systemically minimize the ambiguity in setting the milling regions of all FIB processes. Second, the end-point of each FIB process is controlled by pre-defined time instead of SEM monitoring. Due to the lack of in-situ monitoring by SEM, the second point might cause serious deviation of the lamella thickness. However, no systematic study has explored the limitation of such a simplified design of the final-thinning process. Considering the significant benefits of precision and repeatability by robot scientists, we expect that a combination of (i) an automatic correction of the milling position and (ii) a systematic optimization of the FIB parameters in the final-thinning process can realize repeatable preparation of 50-nm-thick high-quality lamellae.

Full automation of the lift-out process is another challenge of the robotic FIB. A pioneering work by Van Leer et al.^30^ reported a semi-automated FIB. In the report, semi-automatic software controls the rough-milling and the final-thinning process according to user-defined parameters. The software also controls the lift-out process by image recognition but always requires double-confirmation of the image recognition by an operator. This limits the practical throughput of preparing STEM lamellae and makes it difficult to study systematically the FIB parameters in the final-thinning process. We also note that the experiments in Ref.^30^ used 30 kV thinning and the final-polishing at 5 kV since the automation software restricts only one acceleration voltage in the final-polishing step. The resulting damage due to the final polishing at 5 kV should be hardly acceptable for modern aberration-corrected STEM.

Here, we demonstrate that a fully robotic FIB system can prepare high-quality 50-nm-thick lamellae using the latest automation software, which includes full automation of the lift-out process and provides multiple steps of low-kV polishing in the final-thinning process. First, we show that the robotic FIB is now able to prepare lamellae with a high success rate, where the final-thinning process has 30 kV-thinning and 5 kV- and 2 kV-polishing. Upon the robust automation, we systematically explored the optimal FIB parameters of single crystal silicon. We found that the over-tilt angle affects the quality of Si lamellae and the optimal angle at 2 kV-polishing was 1.6°, 3–4 times shallower than a conventional value. To confirm the statistical repeatability of the robotic FIB, we evaluated three Si lamellae prepared by the robotic FIB with the same set of the FIB parameters. The three Si lamellae have almost the same profile of thickness and the thickness at the bottom edge was 40–50 nm. Aberration-corrected STEM evaluated the Si lamellae, all showing atomic-level images with 55 pm resolution. Hence, we experimentally demonstrate that the robotic FIB can prepare high-quality 50-nm-thick lamellae for aberration-corrected STEM, where the repeatability of the resolution and the lamella thickness is also achieved as a significant benefit of robot scientists. We also demonstrate that the robotic FIB can prepare high-quality lamellae of SrTiO_3_ and sapphire. These results suggest that the robotic FIB system may be applicable for a wide range of materials. The throughput of the automatic fabrication of lamellae was typically an hour per lamella. Our robotic FIB system will pave the way for the operator-free, high-throughput, and repeatable fabrication of the high-quality lamellae for aberration-corrected STEM analysis.

## Results and discussion

### Preliminary set-up for the automation software

For robotic FIB operation, we used a focused-ion-beam/scanning electron microscope (FIB/SEM) DualBeam system (Helios 5 UX, Thermo Fisher Scientific) with a new automation software package (AutoTEM 5, Thermo Fisher Scientific). The automation software is a new version of AutoTEM 4, which was used in a pioneering work of a semi-automated FIB system^30^. AutoTEM 5 covers all of the automation functions in rough-milling, lift-out, and the final-thinning process. The final-thinning process in AutoTEM 5 is composed of 30 kV-thinning, one low-kV polishing, and one lowest-kV polishing. We selected 5 kV-polishing and 2 kV-polishing in this study.

Before the automation starts, a user loads the bulk sample and 3-mm TEM grids into the FIB/SEM chamber (Supplementary Fig. S1). Then, the automation software requires input data of the chunk position and its orientation in the FIB image. In this study, we manually aligned the orientation of the rough-milling region so that we can extract Si chunks with its zone axis of [110]. We registered the chunk position randomly in the FIB image. Next, the automation software requires the parameters of the rough-milling process. The parameters include the selection of the material of the protective layer, the height of the protective layer, and the dimension (width, depth, and thickness) of the chunk. After the parameters of the rough-milling process are defined, the automation software further requires the grid position in a TEM grid to which the extracted chunk is transferred in the lift-out process. In this study, we chose a side position of a pole in a Cu grid so that we can minimize the redeposition from the Cu grid. Each Cu grid has four poles. We also limited one lamella at one pole of the Cu grid to avoid unintended irradiation of Ga-FIB to other lamellae.

Finally, the automation software requires a set of FIB parameters in the final-thinning process. The process is composed of 30 kV-thinning (multiple steps of thinning with different FIB current), one low-kV polishing, and another lower-kV polishing. For each FIB process, a user can set the FIB current, the target thickness, and the over-tilt angle. The “target” thickness is defined as the gap between the two milling boxes minus the size of the FIB probe (see also Supplementary Methods). Thus, the target thickness is a nominal value not a physical observable. Two low-kV polishing processes follow after the 30 kV-thinning. In each low-kV process, the user can set the acceleration voltage of the FIB. In this study, we chose 5 kV and 2 kV as the low-kV polishing.

The automation software can manage a batch experiment of multiple fabrications of lamellae. In our study, we fabricated 10 lamellae from Si in one batch experiment over a night. In the multi-site lamellae preparations, a user preliminary inputs the combination of (the chunk position/orientation, the rough-milling parameters, the grid position, the final-thinning parameters) for every lamella. When the preparation of one lamella is completed, the automation software starts the preparation of the next lamella by following the pre-defined parameters. Next, we explain the detailed workflow of our experiment using the robotic FIB.

### Workflow and robustness of the robotic FIB

Figure 1 summarizes how the robotic FIB prepared Si lamella. In the rough-milling process, FIB was used to deposit carbon protective layer with its height of 1.3 μm and cut the chunk with its dimension of 12 μm by 5.0 μm by 2.4 μm (Fig. 1a). Then, an in-situ manipulator automatically approached the chunk and lifted out the chunk from the bulk Si (Fig. 1b). The chunk was transferred to the pre-defined grid position and welded with the Cu grid (Fig. 1c). See Ref.^30^ about the detailed algorithm to calculate the manipulator position in the FIB/SEM microscope. After FIB cut the chunk from the manipulator, the manipulator was retracted. Then, the final-thinning process followed as shown in Fig. 1d–f.

In our study, the final-thinning process is composed of 30 kV-thinning, 5 kV-polishing, and 2 kV-polishing. In the final-thinning process, FIB repeatedly scans the X-shaped alignment marker and corrects the position of the lamella by calculating the cross-correlation between the original image and the present image (Fig. 1d). This automatic correction enhances the precision of the milling box of FIB. The end-point of the FIB thinning/polishing was controlled by time, which is calculated from the target thickness and the width and depth of the thinning region. In the 30 kV-thinning process, we set as the FIB current gradually decreased from 2.4 nA to 26 pA. We also note that the automation software thinned only the “window” region from the middle of the 30 kV-thinning process (Fig. 1d), where the width of the window is also a user-chosen parameter. We set the window width as 2.4 μm in this study. The target thickness of the window in the 30 kV-thinning process is set at 200 nm. After the 30 kV-thinning, FIB automatically changed its acceleration voltage down to 5 kV and 2 kV to polish the window region (Fig. 1e,f). The FIB current was 63 pA at 5 kV and 66 pA at 2 kV. The target thickness of each polishing process was 120 nm at 5 kV and 60 nm at 2 kV. Even when the FIB acceleration voltage was set at 2 kV, our automation software reliably recognized the alignment marker and conducted the 2 kV-polishing process successfully. Note that the SEM images in Fig. 1d–f were manually acquired for references.

Table 1 summarizes the success rate of the robotic FIB experiments for preparing lamellae. In all of our experiments of Si samples (N = 30), the robotic FIB completed all the processes of rough-milling, lift-out, and the final-thinning (including 2 kV-FIB polishing). We also applied the robotic FIB to SrTiO_3_ and sapphire in the later part of this study, where the FIB parameters were similar to those for Si. For both materials, the robotic FIB system also completed all the processes except for one failure in a SrTiO_3_ sample. The success rates of Si, SrTiO_3_, and sapphire were 100%, 95%, and 100%, respectively, in our experiments. After completing the robotic FIB operations, all the lamellae were evaluated by STEM and all showed atomic-level resolution. These results confirm the robustness of our robotic FIB operation even when the material is an insulator. Moreover, the throughput of the automatic preparation of each lamella was typically 1 h per lamella, including all the processes from rough-milling to the final-thinning. Thus, we demonstrate that our robotic FIB can work robustly as a high-throughput and operator-free system. Based on the robust platform of the robotic FIB, we focus on the quality of the lamellae and the quantitative repeatability.

**Table 1:** Summary of the robotic FIB experiments.

	Si	SrTiO_3_	Sapphire
# of experiments	30	20	2
# of successful fabrications	30	19	2
Atomic-level STEM images	30	19	2
Success rate	100%	95%	100%
Throughput of fabrication (minutes per lamella)	50	60	80

The number of experiments includes preliminary tests of fabricating lamellae to explore FIB parameters. The successful fabrication counts the number of the experiments that completed all the automation processes (including 2 kV-FIB polishing). One fabrication of SrTiO_3_ lamella failed in the lift-out process. All the lamellae in “successful fabrications” were evaluated by STEM and yielded atomic-level STEM images. Throughput of fabrication is the duration that the automation software spent in rough-milling, lift-out, and the final-thinning processes of each STEM sample. This value is a typical one and excludes the processing time of loading bulk samples and setting the automation software.

### Optimizing the final-thinning process for Si

**Figure 2:**
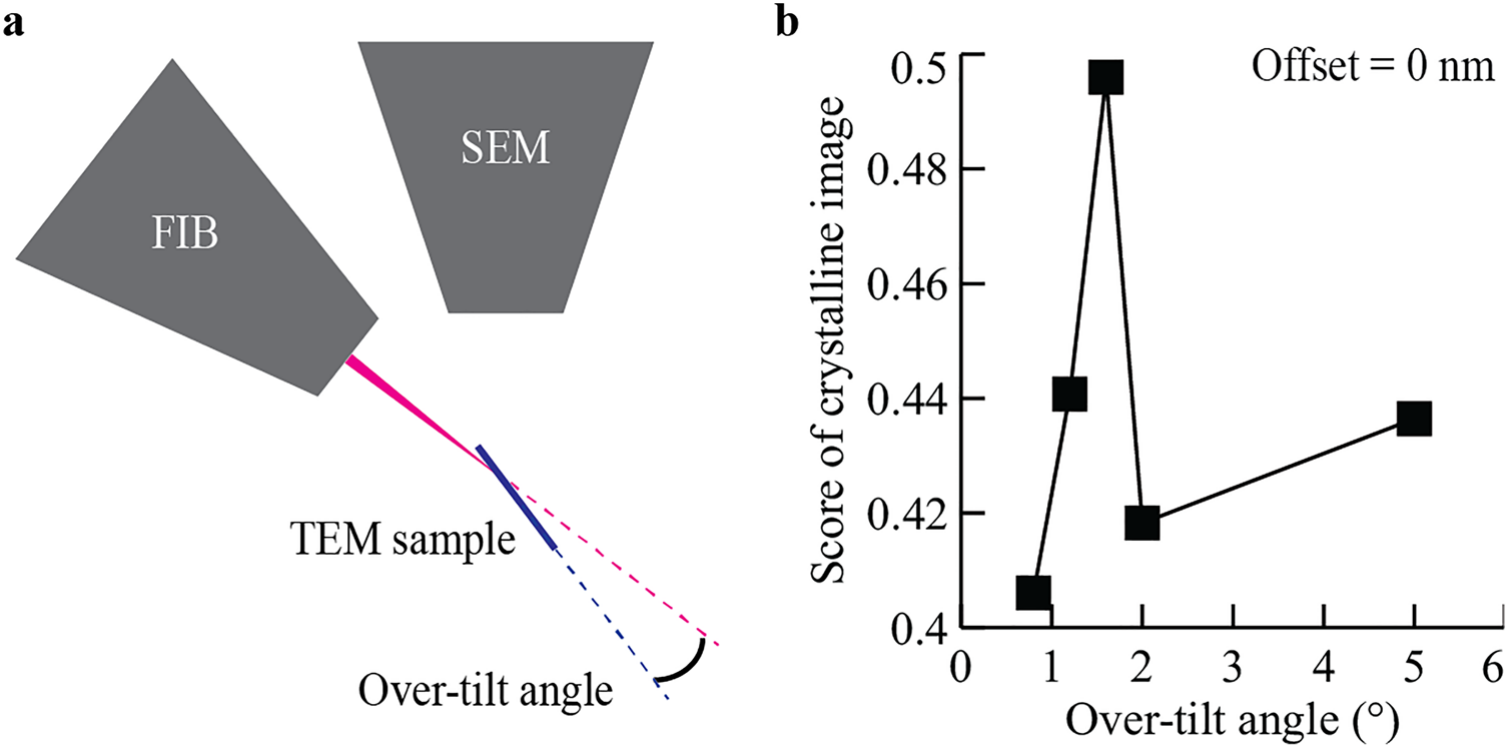
Optimizing FIB parameters for Si. (**a**) A side-view diagram of the over-tilt angle, which is the angle between the plane of the TEM sample and the ion beam. (**b**) A plot of the score of the crystalline image as a function of the over-tilt angle used for the 2 kV-FIB polishing step. We prepared five lamellae, using over-tilt angles of 0.8°, 1.2°, 1.6°, 2.0°, and 5.0°, respectively. For each lamella, the score of the crystalline image was calculated from the high-resolution STEM images (see “Methods” section and Supplementary Fig. S3).

The quality of an atomic-level STEM image primarily depends on the final-thinning process, as previous studies have highlighted^12–14^. It is generally agreed that 30 kV-FIB damages a lamella from its surface to a depth of the order of 10 nm^13,31^. Thus, a cleaning step using low-kV FIB (2 kV or below) is important for reducing the surface damage layer to form a high-quality sample suitable for atomic resolution STEM imaging^12–14^. At low kV, blurring of the FIB image makes it difficult to accurately select the milling location (Fig. 1f). This limitation is typically overcome by using sample over-tilt, so that approximate positioning of the low-kV milling region is sufficient (Fig. 2a). As the over-tilt angle becomes large, Ga ions are implanted into the STEM sample. Furthermore, a high over-tilt angle creates a thickness gradient from the top of the sample (protective layer) to the bottom (edge), which limits the thin area within the sample available for high-quality atomic-level STEM imaging. Hence, the over-tilt angle is selected as a compromise between overcoming FIB blurring, degree of Ga implantation, and sample thickness uniformity.

Although the over-tilt angle may affect the quality of the resulting STEM image, a systematic study to determine the optimal over-tilt angle has not been reported previously. To maximize the quality of the sample surface, we study the impact of over-tilt angle on STEM images. We prepared lamellae from single crystal Si with the 2 kV-FIB over-tilt angle ranging from 0.8° to 5.0°. The resulting lamellae were evaluated by conventional STEM (see “Methods” section). We shortly checked that the resulting lamellae show no curtaining effects on its surface (Supplementary Fig. S2). To compare the impact of different over-tilt angles in STEM analysis, we acquired atomic-level high-angle annular dark-field (HAADF) images in a region near the top protective layer for each Si lamella (Supplementary Fig. S3). To quantify the quality of a crystalline image, we introduce a scoring function $f\left(I\right)$, where $I$ is an atomic-level STEM image. In calculating $f\left(I\right)$, the atomic-level image is separated into a periodic image (i.e., crystalline image) and the rest. $f\left(I\right)$ returns the fraction of the crystalline image in the total image. Thus, $f\left(I\right)$ is an order parameter ranging from 0 (complete amorphous) to 1 (complete crystal). See the detailed definition of $f\left(I\right)$ in Supplementary Fig. S3 and “Methods” section. In Fig. 2b, we compute $f$ for the STEM images of the Si lamellae that FIB polished at a different over-tilt angle at 2 kV. The score of the Si crystalline image is highest when using an over-tilt angle of 1.6°.

The optimal over-tilt angle in our study is much lower than that of previous reports (5°–7° at 2 kV)^14^. We note that the FIB/SEM system type used is different from previous studies. Our latest FIB/SEM should have a sharper low-kV FIB beam profile and might shift the trade-off between blurring and ion damage towards a lower over-tilt angle. The targeted sample shape is also different. We aimed to achieve samples with uniform thickness in the lamella plane whereas the previous studies aimed to create wedge-shaped lamellae thinnest at the bottom edge. In other words, the optimal over-tilt angle may depend on the FIB/SEM tool used and the target profile of the lamellae (parallel-sided or wedge-shaped). In our study, we established the optimal over-tilt angle for single crystal Si, at least for our evaluation criteria.

After optimizing the over-tilt angle at 1.6°, the thickness of the Si lamella was fine-tuned. We note again that the “target” thickness is the gap between the two milling boxes minus the size of the FIB probe. So, the target thickness is not equal to the actual thickness of the lamella. To correct the difference, a user can set another parameter of the milling position offset in the FIB polishing process (see Supplementary Fig. S4 and Supplementary Methods). We tested several values of the milling offset and chose the optimal value as the lamella has the lowest but non-zero thickness in the bottom edge (Supplementary Fig. S4). Next, we characterize the quality and repeatability of the optimized Si lamellae.

### Characterizing optimized Si lamellae by aberration-corrected STEM

**Figure 3:**
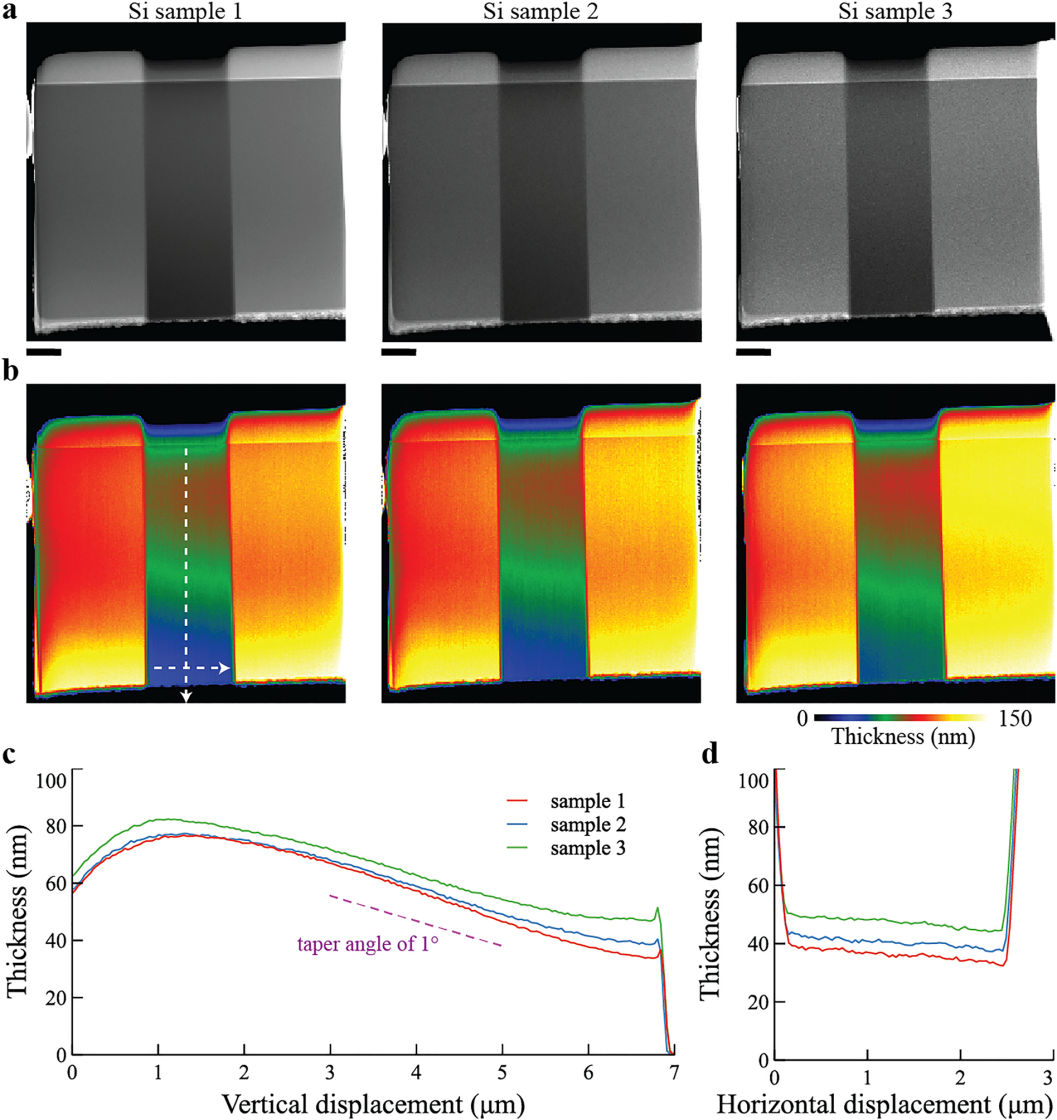
Sample shape and thickness repeatability of robotically prepared Si lamellae. (**a**) HAADF-STEM images of each sample. The three lamellae were fabricated using the Si-optimized settings. (**b**) Thickness maps generated from EELS spectrum imaging. (**c**) Thickness profiles in the vertical direction (from the top protective layer to the bottom edge). The dashed line shows a 1.0° taper angle. (**d**) Thickness profiles horizontally across the thin window near the bottom edge. The scale bar is 1 μm.

We prepared three Si lamellae by robotic FIB with the optimal settings for silicon (Fig. 3a). Figure 3b shows thickness maps of the three lamellae acquired using electron energy loss spectroscopy (EELS). Measuring vertically from the top protective layer towards a bottom edge, the sample thickness increases rapidly from 60 to 80 nm and then gradually decreases to ∼ 40 nm. The taper angle between the faces is ∼ 1° (Fig. 3c). In the region near the bottom edge, the thickness across the window width is almost constant at 40–50 nm (Fig. 3d). This sample thickness is suitable for high-quality atomic-level STEM imaging. And the thickness variation among the three samples was only 10 nm (Fig. 3c,d). We emphasize that this profile and thickness repeatability is a notable benefit of robotic experimentation and that such repeatability is important for direct comparison between STEM images from multiple samples.

**Figure 4:**
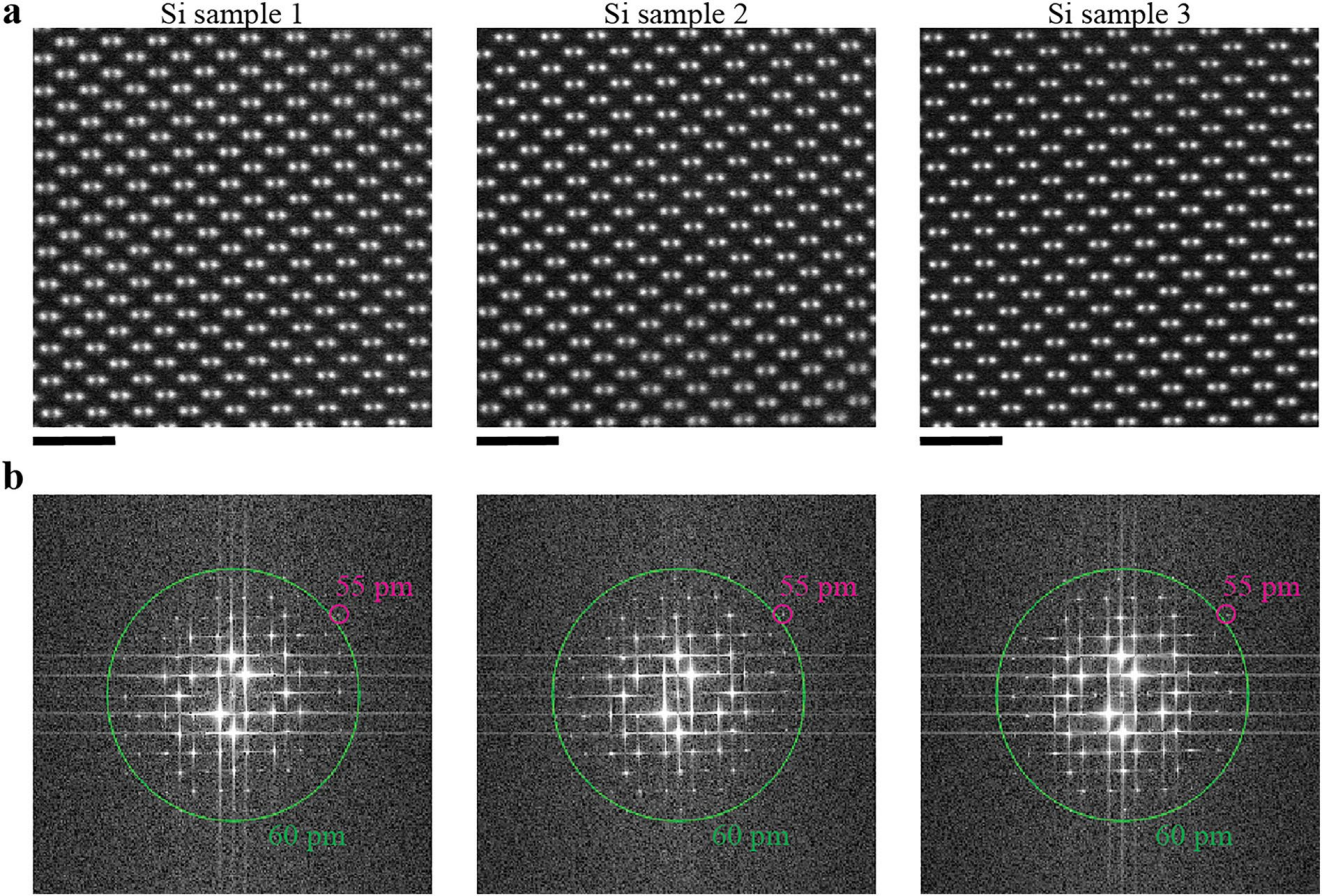
Aberration-corrected STEM imaging of the three Si lamellae. (**a**) Atomic-level HAADF-STEM images of each lamella along the [110] direction acquired by aberration-corrected STEM (same samples as Fig. 3). Images were acquired near the thin bottom edge of each sample. Equivalent HAADF-STEM images from near the top protective layer are shown in Supplementary Fig. S5. (**b**) FFT patterns for each image. Magenta circles indicate FFT reflections at 55 pm resolution. Green circles correspond to 60 pm resolution. The scale bar is 1 nm.

We evaluated the suitability of the Si lamellae for aberration-corrected STEM imaging (see “Methods” section). Figure 4a shows atomic-level HAADF-STEM images from regions near the sample edge. The Si dumbbell structure is visible in all three images. Fast Fourier transforms (FFTs) confirm 55 pm spatial resolution in all the lamellae (Fig. 4b). We also acquired high-resolution HAADF-STEM images in regions near the top protective layers, yielding spatial resolution of 55 pm (Supplementary Fig. S5). We note that the guaranteed resolution of our STEM is 50 pm when using 300 kV electrons. So, our robotic FIB system realized repeatable fabrication of high-quality lamellae that yield deep sub-angstrom resolution, close to the specified resolution of the latest aberration-corrected STEM instruments.

### Robotic fabrication of high-quality lamellae from SrTiO_3_ and sapphire

We next evaluated robotic fabrication using other materials. As shown with Si, the over-tilt angle used for 2 kV cleaning affects the quality of STEM samples. Whereas the final sample thickness depends strongly on the milling rate of each material and thus the procedure needs to be adjusted, validating that the 1.6° over-tilt angle is an effective setting for a range of other materials is also of great importance. This validation matters especially for hard materials, such as SrTiO_3_, sapphire, diamond, and steel, which are used in various functional materials^16,18,21,22^. We thus tested the robotic sample fabrication on SrTiO_3_ and sapphire, keeping the over-tilt angle for 2 kV-FIB unchanged at 1.6°.

We modified the Si-optimized procedure for SrTiO_3_ and sapphire as follows: First, as both materials are insulators (unlike silicon). To suppress charging effects during FIB imaging and milling, we sputtered chromium on the surface of the bulk samples before loading them into the FIB/SEM chamber. Second, to adjust for the different milling rates of these materials, we performed milling rate tests on bulk crystals using 30 kV FIB (Supplementary Fig. S6). Counting the milling rate of Si as 1, the milling rates of SrTiO_3_ and sapphire are slower at 0.5 and 0.3, respectively. So, we scaled all durations of FIB processes as 3 times and 4 times longer than ones of Si-optimal conditions for SrTiO_3_ and sapphire, respectively. We also changed the depth of the chunk as 3 µm to shorten the fabrication time. With this adjusted recipe, our robotic FIB system showed a high success rate of fabricating lamellae from both SrTiO_3_ and sapphire (Table 1). The robotically fabricated lamellae were evaluated by STEM imaging and all showed atomic resolution. And third, we tuned the sample thickness by adjusting the FIB milling offset in the same way as shown in Supplementary Fig. S4, while keeping the over-tilt angle (of 2 kV-FIB milling) constant at 1.6°.

**Figure 5:**
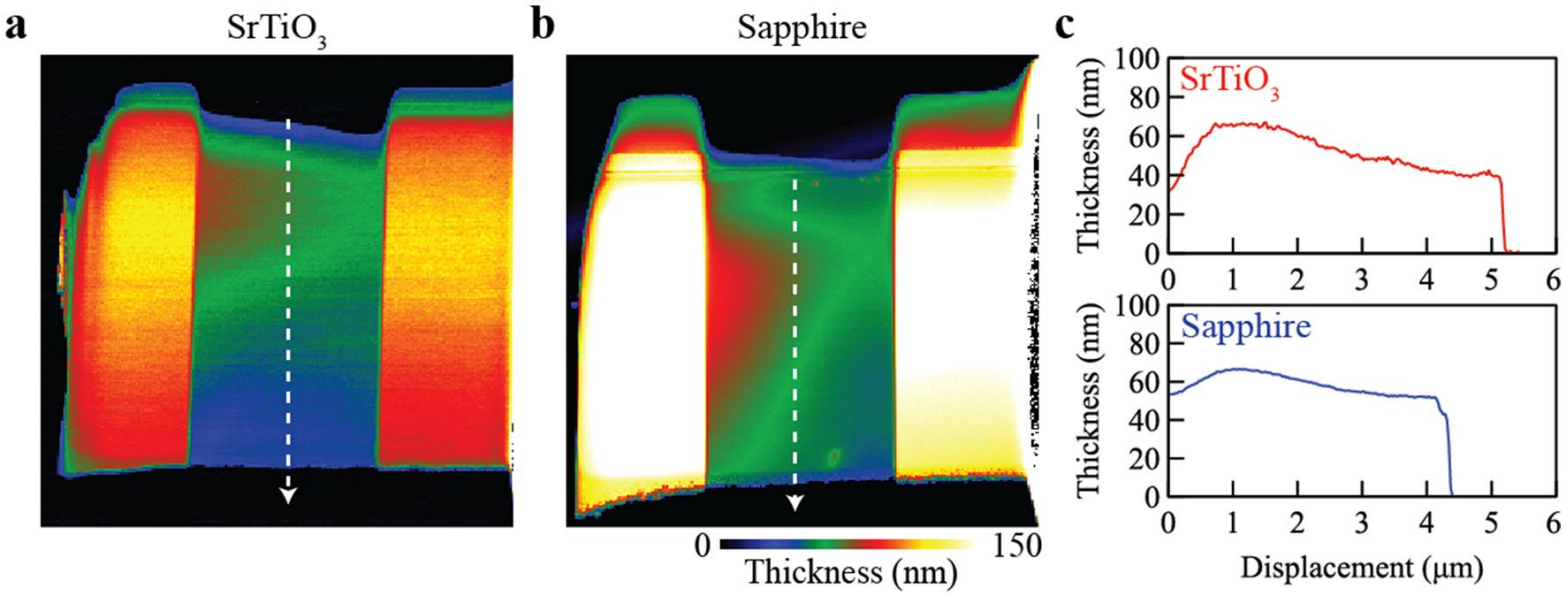
Sample thickness of SrTiO_3_ and sapphire lamellae prepared by the optimized robotic FIB. (**a,b**) Thickness maps from low loss EELS spectrum imaging of SrTiO_3_ and sapphire, respectively. (**c**) Thickness profiles from the top protective layer to the bottom edge.

**Figure 6:**
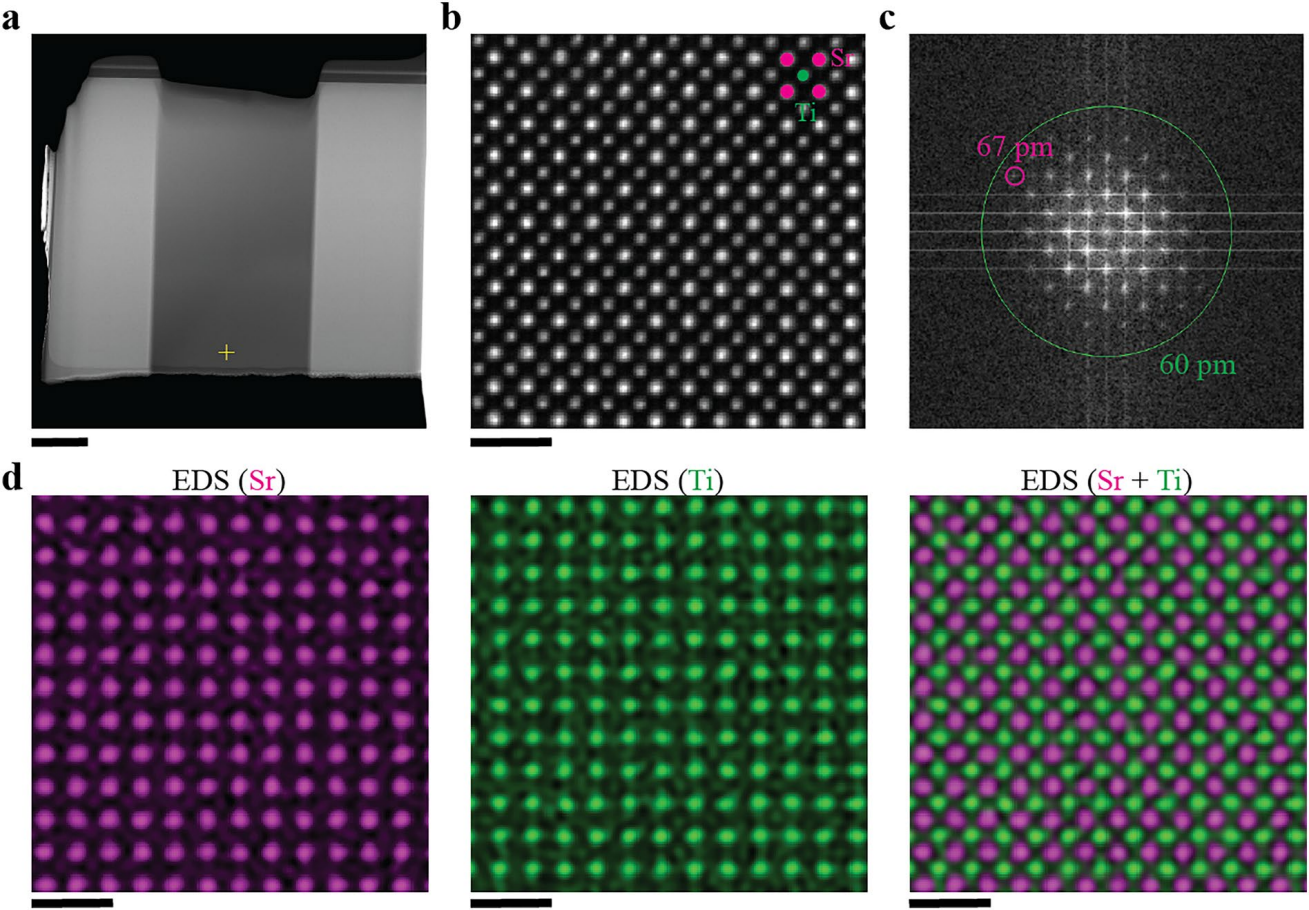
Aberration-corrected STEM results of a robotically prepared SrTiO_3_ lamella. (**a**) A HAADF-STEM image of the entire SrTiO_3_ lamella. (**b**) An atomic-level HAADF-STEM image along the [100] direction. The image was acquired near the thin bottom edge of the SrTiO_3_ lamella (marked + in (**a**)). (**c**) FFT pattern of (**b**). The magenta circle indicates an FFT reflection at 67 pm resolution. (**d**) Atomic-level EDS maps for Sr (left), Ti (center), and Sr combined with Ti (right). The scale bar in (**a**) is 1 μm, that in (**b,d**) is 1 nm.

**Figure 7:**
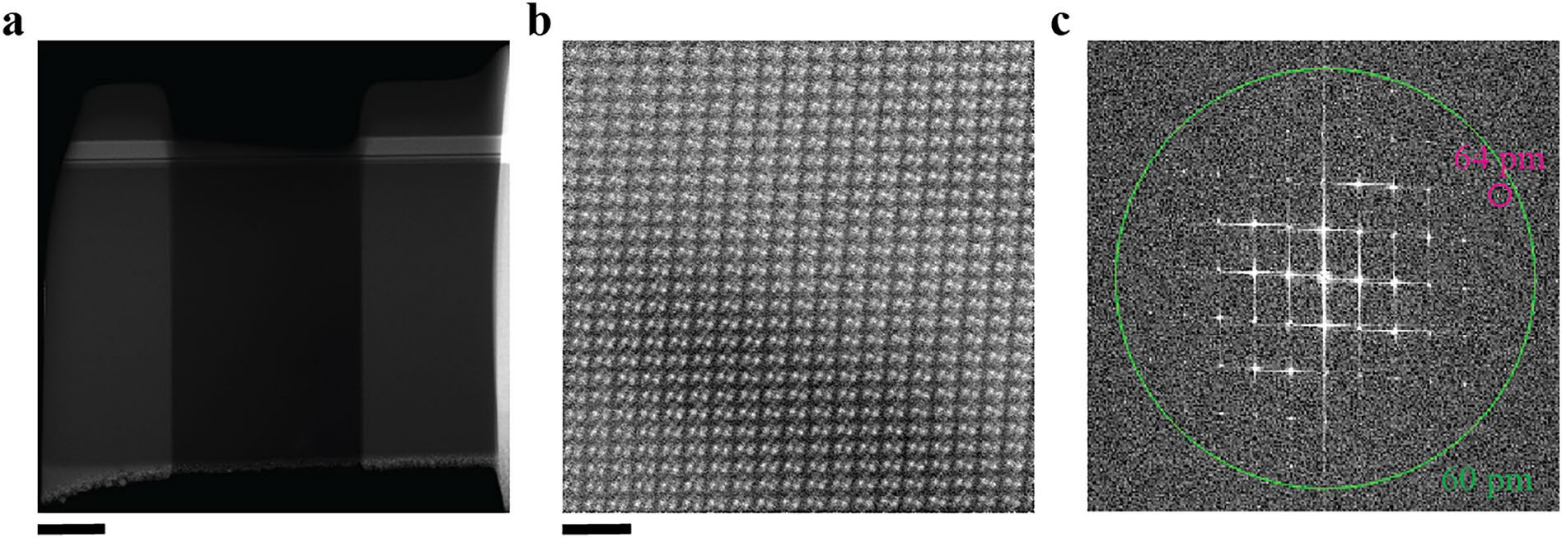
Aberration-corrected STEM imaging of a robotically prepared sapphire lamella. (**a**) A HAADF-STEM image of the entire sapphire lamella. (**b**) An atomic-level HAADF-STEM image near the edge of the sapphire sample. See Ref.^32^ for the atomic model of this material. (**c**) FFT pattern of (**b**). Scale bars in (**a,b**) correspond to 1 μm and 1 nm, respectively.

We characterized the resulting lamellae by aberration-corrected STEM. The profile and thickness of both the SrTiO_3_ and sapphire samples were similar to that of Si (see Fig. 5). All lamellae had a thickness of ∼ 50 nm near the bottom edge. Figure 6 summarizes the aberration-corrected STEM results of the SrTiO_3_ lamella. A high contrast HAADF-STEM image in Fig. 6b clearly shows the position of the Sr and Ti atomic columns, and with spatial resolution of 67 pm (Fig. 6c). Energy-dispersive X-ray spectroscopy (EDS) mapping of the SrTiO_3_ sample also clearly shows the atomic columns of Sr and Ti (Fig. 6d). These results suggest that the over-tilt angle of 1.6° in the Si-optimal recipe is also suitable for SrTiO_3_. Figure 7 shows the aberration-corrected STEM results of the sapphire lamella. The STEM image visualizes Al atoms with 64 pm resolution, showing apparent similarity with a previous report^32^. Since the atomic-level STEM image in Fig. 7b includes a dark band, the FIB parameters can be tuned better for sapphire. But we may say that the Si-optimal parameters can work for sapphire at least as an initial experiment.

Throughout the experiments of preparing SrTiO_3_ and sapphire lamellae, we show that the over-tilt angle of the Si-optimal recipe can be suitable for a wide range of materials, which may expand the potential applications of the robotic FIB among various single crystals and nanostructures grown on the single crystal substrates^15,18,20,33,34^.

### Throughput of robotic fabrication of high-quality lamellae

Regarding the throughput, the robotic fabrication of each lamella took approximately 50 minutes (Si), 60 minutes (SrTiO_3_), and 80 minutes (sapphire) in our study (see Table 1). Moreover, the automation software can run sequential fabrications of multiple lamellae without the attendance of an operator. The robotic FIB system may increase the total throughput of fabricating high-quality lamellae several times relative to that of the current manual FIB procedure.

We especially expect that the robotic FIB will play key roles when the aberration-corrected STEM analysis is performed in a study of combinatorial chemistry. A combinatorial method can synthesize a bulk material that has compositional gradients, where a position in the bulk corresponds to its composition^11,33–36^. In recent studies of combinatorial chemistry, aberration-corrected STEM is sometimes used but the number of lamellae (i.e., the number of studied compositions) is very limited^34,36^. If the robotic FIB is used, preparing high-quality lamellae at more than ten positions (i.e., more than ten compositions) may become realistic, and aberration-corrected STEM analysis will provide atomic-level insights more in a study of combinatorial chemistry.

### Current limitations of the robotic FIB and key challenges in the future

This study has demonstrated a robotic FIB platform as an operator-free, high-throughput, and repeatable method to prepare 50-nm-thick lamellae for aberration-corrected STEM from standard single crystals. To broaden the practical applications of the robotic FIB in the future, we discuss the current limitations of the robotic FIB system and the prospective challenges.

**Figure 8:**
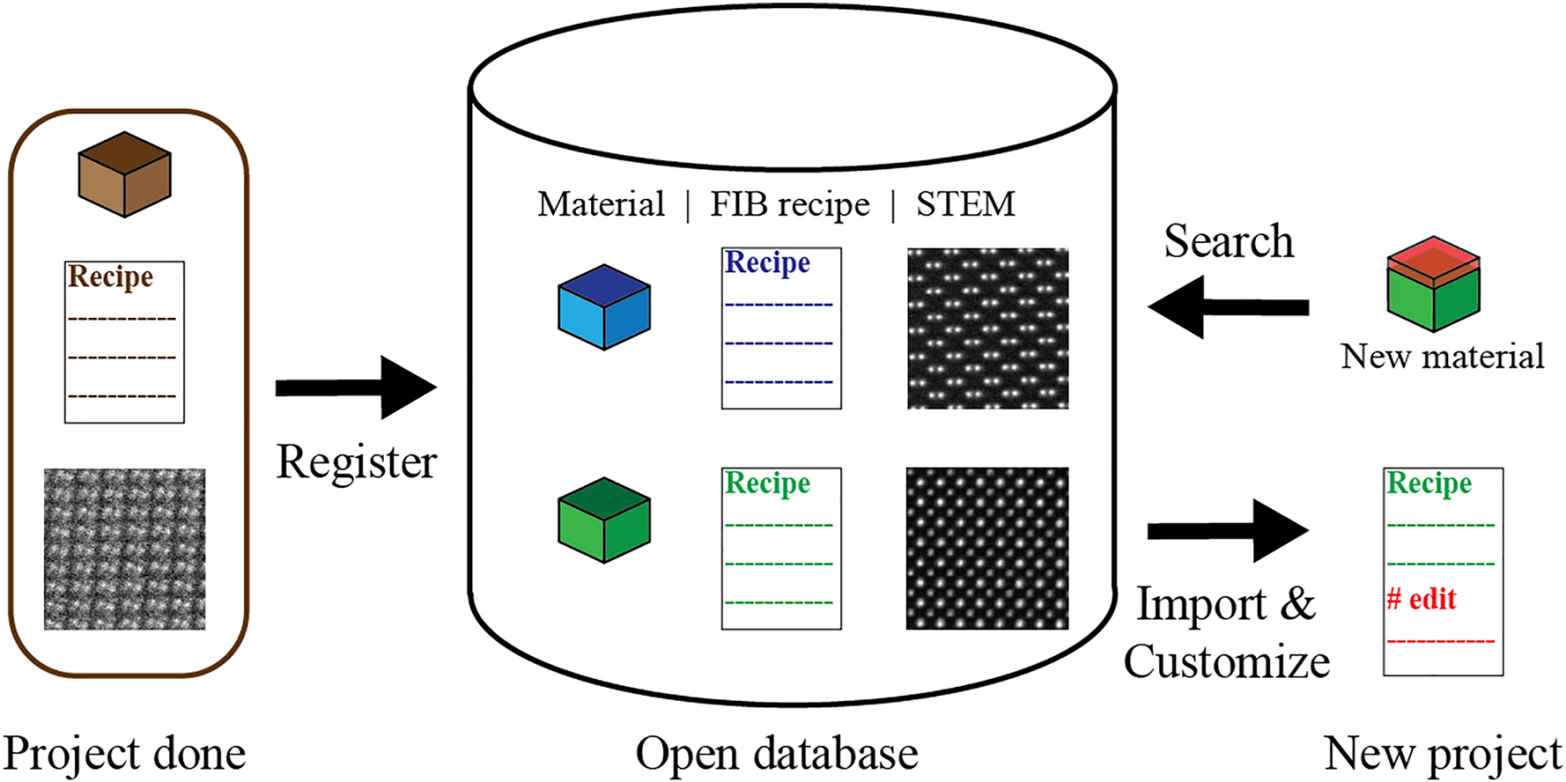
A conceptual schematic of an open database of FIB recipes. An open database stores data of material information (e.g., compositions), the digitalized recipe of the robotic FIB, and its STEM results. Any user can register new results. According to the material information, any user can search and import an FIB recipe that is suitable for the targeted material from the database. The user can further adjust the FIB recipe for the targeted material. Hence, the open database accelerates exchanging the knowledge of FIB in the whole community of materials scientists.

A large variety of sample types in materials science is one of the primary challenges of the robotic FIB. Although the operation of the robotic FIB depends on a set of the digitalized parameters (a “recipe”) for each material, we currently have tuned the FIB recipes for only standard materials. Thus, further experiments to expand the variety of the FIB recipes (for various material types) are highly needed. Here, we emphasize the significant benefit of the digitalized protocol. Unlike a manual FIB, the robotic FIB stores all knowledge as a set of digital parameters. The digitalized protocol allows researchers to share, search, import, and customize the existing recipes. If a microscopy society organizes an open database that lists various FIB recipes tagged with the material information and the STEM results, the future researcher will be able to save the time to find the suitable FIB parameters (see Fig. 8).

Another key challenge of the robotic FIB is to improve the way to control the end-point of the final-polishing step. Currently, the robotic FIB does not monitor the SEM in the final-thinning process. The resulting thickness (i.e., the end-point of the final-polishing step) is mainly controlled by the milling-offset parameter as shown in Supplementary Fig. S4. The experiments for fine-tuning take some time. In a manual FIB, an operator usually determines the end-point by monitoring SEM during the FIB polishing. Typical end-point criteria in a manual FIB are the height of the residue of the top protective layer or the transparency of the lamella interpreted from secondary-electron SEM images^37^. It may not be difficult to implement an automatic loop of acquiring SEM images on the robotic FIB during the final polishing. In contrast, automatic interpretation of the SEM images and automatic determination of the end-point of the final-polishing should be a key challenge to improve the controllability of the resulting thickness and make the robotic FIB more user-friendly.

This study has targeted robotic fabrication of 50-nm-thick lamellae as a common requirement of aberration-corrected STEM. But recent advances of aberration-corrected STEM often require 10-nm-thick lamellae due to the assumption of weak phase objects. The advanced STEM methods include annular bright field imaging^38^, differential phase contrast (DPC) imaging^39^, integrated/differential DPC (iDPC/dDPC) imaging^40–43^, and EELS mapping with high-energy resolution^44–48^. How can the robotic FIB contribute to preparing such extremely thin lamellae? When preparing the extremely thin lamellae, the final-polishing process must use the lowest acceleration voltage to minimize the thickness of the damage layer down to a sub-nm range^12–14^. Although a standard FIB system can select 500 V as the lowest acceleration voltage, the FIB images at 500 V are too blurred and the automation software can hardly recognize the alignment marker in the FIB image. This primarily limits a fully robotic preparation of high-quality 10-nm-thick lamellae and thus requires extra manual polishing by FIB at 500 V or an external Ar-ion milling tool^16,17,22,41,49^. Even in such a hybrid methodology, the robotic FIB may play a wide role to prepare 50-nm-thick lamellae that have thickness uniformity in the lamella plane (see Fig. 3), which might enhance both throughput and success rate of preparing 10-nm-thick lamellae.

Since this study has focused on single crystals, we randomly set the rough-milling positions, where the orientation of the lamella was properly aligned with the orientation of the bulk single crystal. When a bulk material has inhomogeneities (e.g., polycrystalline materials), the linkage between atomic-level STEM analysis and mesoscopic-level information of SEM, EDS, and electron backscattered diffraction (EBSD) is of great importance to understand the structure–property relationships in multiple length-scales^10^. Thus, a linkage of position/orientation data between SEM-based analysis and the robotic FIB platform will become important. Moreover, machine-learning for the high-throughput analysis of poly-crystalline microstructures from SEM and EBSD data has been intensively studied^50,51^. So, the future integration of the high-throughput SEM/EBSD analysis with the high-throughput STEM analysis is highly desired for a systematic and multi-scale structural analysis of polycrystalline materials.

## Conclusion

In conclusion, this study demonstrates a robotic FIB system to fabricate high-quality lamellae from a wide range of materials. We systematically optimized the FIB parameters of the final-thinning process for single crystal Si and adjusted the Si-optimal parameters to SrTiO_3_ and sapphire. The resulting lamellae of Si, SrTiO_3_, and sapphire were evaluated by aberration-corrected STEM and yielded a spatial resolution of 55 pm (Si), 67 pm (SrTiO_3_), and 64 pm (sapphire). These results confirm that the quality of robotically-prepared lamellae can meet the requirements of aberration-corrected STEM. Moreover, our results show that the resolution and sample thickness can be repeatable, which is a significant benefit of robotic experimentation. The throughput of the robotic FIB was approximately an hour per lamella, which is several times higher than that of manual FIB processes. So, our demonstration of the robotic FIB system will pave the way for the operator-free, high-throughput, and repeatable fabrication of the high-quality lamellae suitable for aberration-corrected STEM analysis.

## Methods

### Focused ion beam system

We used an FIB/SEM (Helios 5 UX, Thermo Fisher Scientific) with the latest automation software (AutoTEM 5, Thermo Fisher Scientific). A motorized manipulator (EasyLift, Thermo Fisher Scientific) is integrated with the FIB/SEM and controlled via AutoTEM 5 software. Detail workflow of AutoTEM 5 is explained in the main text and Supplementary Methods.

### Preparing STEM samples of SrTiO_3_ and sapphire

First, we sputtered Cr on the surface of single crystals of SrTiO_3_ and sapphire to avoid the charging effect. Then, the bulk crystals were loaded into FIB/SEM chamber. FIB parameters of acceleration voltages and currents are identical with the Si-optimal ones, except for the milling duration and the depth of the chunk. In SrTiO_3_ and sapphire, the duration in all FIB processes is elongated from the Si-optimal recipe by a factor of three and four, respectively.

### Conventional transmission electron microscopy

We used a conventional STEM (Talos F200X, Thermo Fisher Scientific) operated at 200 kV for optimizing FIB parameters of Si, SrTiO_3_, and sapphire. The beam current is 50 pA. The convergence angle is 10.5 mrad. Collection angle of HAADF is 28–169 mrad. The specified resolution of the conventional STEM is 160 pm.

### Aberration-corrected transmission electron microscopy

For deep sub-angstrom atomic-level STEM imaging, we used an aberration-corrected STEM (Spectra 300 X-CFEG, Thermo Fisher Scientific) operated at 300 kV. The specification resolution of Spectra 300 is 50 pm at 300 kV. We acquired EDS and EELS mapping by using quadrant EDS detectors (Super-X, Thermo Fisher Scientific) and EELS spectrometer (Continuum ER1065, Gatan) on Spectra 300. For HAADF-imaging and EELS mapping of Si samples, convergence angle and beam current are 26.2 mrad and 50 pA. For HAADF-imaging and EELS mapping of SrTiO_3_ samples, convergence angle and beam current are 30 mrad and 65 pA. For EDS mapping of SrTiO_3_ samples, convergence angle and beam current are 21.4 mrad and 245 pA. For HAADF-imaging and EELS mapping of sapphire samples, convergence angle and beam current are 21.4 mrad and 23.4 pA. Collection angle of HAADF-imaging is 65–200 mrad in all acquisitions. Mean free path (λ) of EELS is estimated as 104 nm (Si), 98 nm (SrTiO_3_), and 115 nm (sapphire). We used OptiSTEM + software (Thermo Fisher Scientific) for acquiring atomic-level STEM images with deep sub-angstrom resolution. The software automatically corrects defocus, twofold astigmatism, coma, and threefold astigmatism at the region of interest. We note that OptiSTEM + minimizes artificial deviations of aligning electron optics, providing quantitative repeatability of deep sub-angstrom resolution to atomic-level STEM imaging. In post-acquisition processing, drift-corrected frame integration is applied for atomic-level images of both HAADF and EDS mapping. For atomic-level EDS mappings, Radial Wiener filter is also applied.

### Computing score of a crystalline image

We quantify the quality of a crystalline image of single-crystal Si by the following procedure. An atomic-level STEM image, ${I}_{0}\left(x,y\right)$, was acquired by conventional STEM (see Supplementary Figs. S2c, S3a as an example). The original STEM image is transformed in reciprocal space as an original FFT pattern (Supplementary Fig. S3a inset). Then, the original FFT pattern is filtered into a crystalline pattern (including only periodic spots) (Supplementary Fig. S3b inset). The crystalline FFT pattern is inverted into the real-space image as a crystalline image, ${I}_{1}\left(x,y\right)$ (Supplementary Fig. S3b). Then, a noise image, ${I}_{2}\left(x,y\right)$, is also extracted as ${I}_{2}\left(x,y\right)={I}_{0}\left(x,y\right)-{I}_{1}\left(x,y\right)$. Instead of simple averaging of image intensity, we calculate the standard deviation of the image intensity because the baseline of image intensity depends on the amplifier settings of every image acquisition. Hence, crystalline and noise scores, ${\sigma }_{1}$ and ${\sigma }_{2}$, are defined as the standard deviation of intensity in the crystalline and noise image, respectively: ${\sigma }_{1}=\sqrt{\langle {{I}_{1}}^{2}\left(x,y\right)-{\langle {I}_{1}\left(x,y\right)\rangle }^{2}\rangle }$ and ${\sigma }_{2}=\sqrt{\langle {{I}_{2}}^{2}\left(x,y\right)-{\langle {I}_{2}\left(x,y\right)\rangle }^{2}\rangle }$,where $\langle \rangle$ averages over $\left(x,y\right)$ in an image. Finally, the score of the crystalline image is computed as $f\left({I}_{0}\right)={\sigma }_{1}/\left({\sigma }_{1}+{\sigma }_{2}\right)$. This order parameter can range from 0 (complete amorphous) to 1 (complete crystal).

## Supplementary Information

Supplementary Information.

## Data Availability

All data needed to evaluate the conclusions in the paper are present in the paper and the Supplementary Information. Upon a reasonable request, corresponding authors can provide the FIB recipes for Si, SrTiO_3_, and sapphire. (Contact H.T.).
