# Supplementary material for: Robotic fabrication of high-quality lamellae for aberration-corrected transmission electron microscopy
Source: Sci Rep. 2021 Nov 3;11:21599. doi: 10.1038/s41598-021-00595-x (PMC8566590; doi:10.1038/s41598-021-00595-x)
Supplement: Supplementary file 1 — Supplementary Information. [file 41598_2021_595_MOESM1_ESM.pdf]

Supplementary Information for

Robotic fabrication of high-quality lamellae for aberration-corrected transmission electron microscopy

Hideyo Tsurusawa*†, Nobuto Nakanishi†, Kayoko Kawano,
Yiqiang Chen, Mikhail Dutka, Brandon Van Leer, Teruyasu Mizoguchi*

†These authors contributed equally to this work.

* Corresponding authors.
Email: Hideyo.Tsurusawa@thermofisher.com, teru@iis.u-tokyo.ac.jp

Supplementary Methods

Supplementary Fig. 1 The sample holder that we used

Supplementary Fig. 2 Results of the Si lamellae by a conventional STEM

Supplementary Fig. 3 Image processing for computing score of a crystalline image

Supplementary Fig. 4 Fine-tuning of the thickness of a Si lamella by milling offset.

Supplementary Fig. 5 Aberration-corrected STEM imaging of Si lamellae near the top protective layer.

Supplementary Fig. 6 Comparing milling rates between Si, $SrTiO_3$, and sapphire.

Supplementary Methods

We explain how AutoTEM 5 software works in detail.

1. Major parameters that a user can choose and tune

AutoTEM 5 stores many parameters in one recipe. Here, we explain major parameters that have a fundamental impact on fabricating high-quality STEM lamellae. We also note how we set the parameters in this study.

1.1. User-chosen parameters in a rough-milling process

To form a protective layer, a user can define deposition material (Pt, C, or W) and the height of the protective layer. AutoTEM 5 automatically calculates the deposition time. Optionally, a user can set an electron-beam deposition before FIB deposition. Then, a user can define the dimension of the chunk (width, depth, and thickness). AutoTEM 5 automatically sets the trench shape whereas the user can change the trench angle and its depth if needed.

To correct the difference in the milling rate of each material, the user can define the correction factor of milling rate that is the relative value to the one of single-crystal Si. This correction factor automatically multiplies the milling durations of all the FIB milling in both the rough-milling and final-thinning processes. For example, we set the correction factor of 3 and 4 in SrTiO and sapphire, respectively.

1.2. User-chosen parameters in a lift-out process

This process has a small number of parameters. A user can define the position of the TEM grid where the TEM chunk is transferred. As reported in ref [1], the user can also define whether the attached destination is the side position of a TEM or the top position (see an example in the

later part of ref [1]). In this study, we selected the side position so that we can minimize the effect of redeposition from the Cu grid.

1.3. User-chosen parameters in a final-thinning process

In AutoTEM 5, the final-thinning process is mainly composed of four steps: step i) thinning of the entire chunk by 30 kV-FIB, step ii) further thinning of the "window" region of the chunk by 30 kV-FIB, step iii) polishing of the window region by low-kV FIB (V_1), and iv) final polishing of the window region by lower-kV FIB (V_2). In step ii), the user can define the width of the window region. In this study, we set the width of the window as 2.4 μm (see Fig. 1d). In the steps of iii) and iv), the user can define the two values of acceleration voltages used in low-kV polishing, V_1 and V_2. In this study, we set V_1 = 5 kV and V_2 = 2 kV.

For every process of FIB thinning / polishing, the user can define FIB current, over-tilt angle, target thickness, and milling offset. For every process of FIB milling, the end-point is determined by the milling time. AutoTEM 5 calculates the milling time of each milling process from the inputs of the target thickness, the width and the depth of the milling region, and the correction of milling rate relative to that of single-crystal Si. See the following section on the definition of the target thickness.

2. Definition of the "target" thickness in AutoTEM 5

Target thickness in AutoTEM 5 is a nominal value, not measured physically. AutoTEM 5 sets the two milling boxes so that the gap between the two boxes is equal to the sum of the target thickness and the probe size of the FIB at the user-chosen acceleration voltage and current. See also the following schematic.

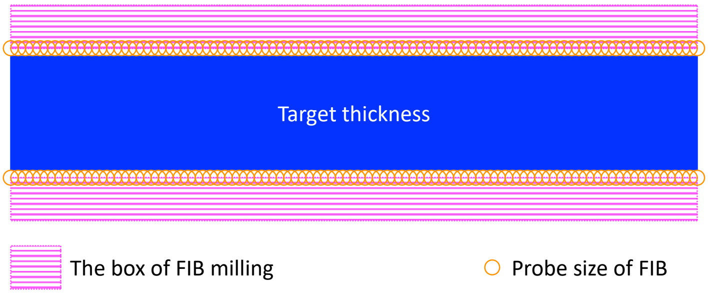

Especially, in the final low-kV polishing, the difference between the “target” thickness and the “actual” thickness has a primary impact on the resulting thickness of the STEM lamella. So, AutoTEM 5 can fine-tune the position of the milling box as an extra parameter of milling offset (see Supplementary Fig. 4).

3. Image recognition of X-shaped alignment marker

X-shaped makers are initially fabricated by FIB deposition on the bulk material and FIB milling (see Fig. 1a). The bigger marker is for aligning the milling positions in a rough-milling process. The smaller marker is for aligning the milling positions in the final-thinning process (see Fig. 1d). To correct the position of FIB milling, AutoTEM 5 repeatedly scans the X-shaped alignment marker. Then, AutoTEM 5 calculates the cross-correlation between the original and the present FIB images. In case of the sample is tilted, the effect of tilt is also considered. From the results of cross-correlation, AutoTEM 5 corrects the position and starts FIB milling. The value of cross-correlation is also used when the FIB focus is aligned.

If automatic fabrication of a lamella fails image recognition, AutoTEM 5 skips the rest process of the lamella. A user can review what process failed in the user interface of AutoTEM 5. If an error occurs in a batch experiment of preparing multiple lamellae, AutoTEM 5 skips the error lamella and automatically starts preparing the next lamella.

Reference

1. Van Leer, B. *et al.* New Workflows Broaden Access to S/TEM Analysis and Increase Productivity. *Micros. Today* **26**, 18–25 (2018).

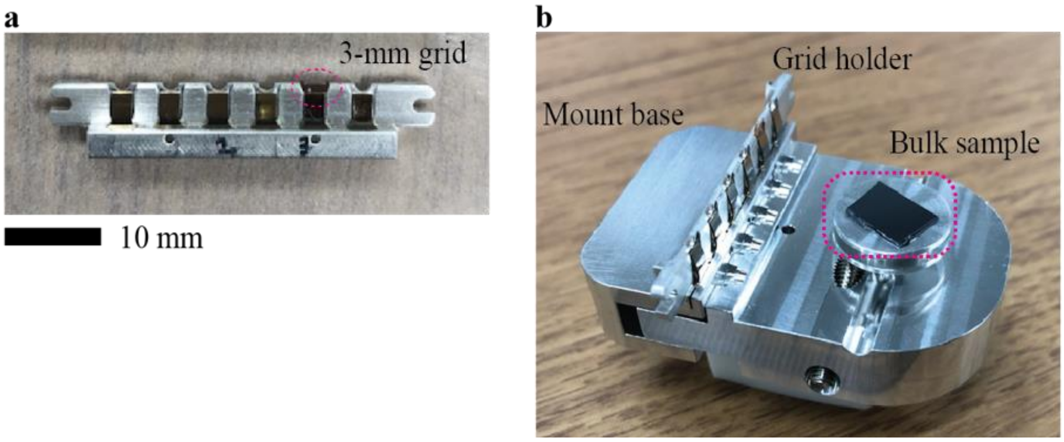

Supplementary Fig. 1 The sample holder that we used. a 3-mm grids are loaded onto a grid holder. Up to 6 grids can be loaded at one time. **b** The grid holder is mounted on a base. A bulk sample is also fixed on the mount base. The mount base is fixed on the stage of the FIB/SEM.

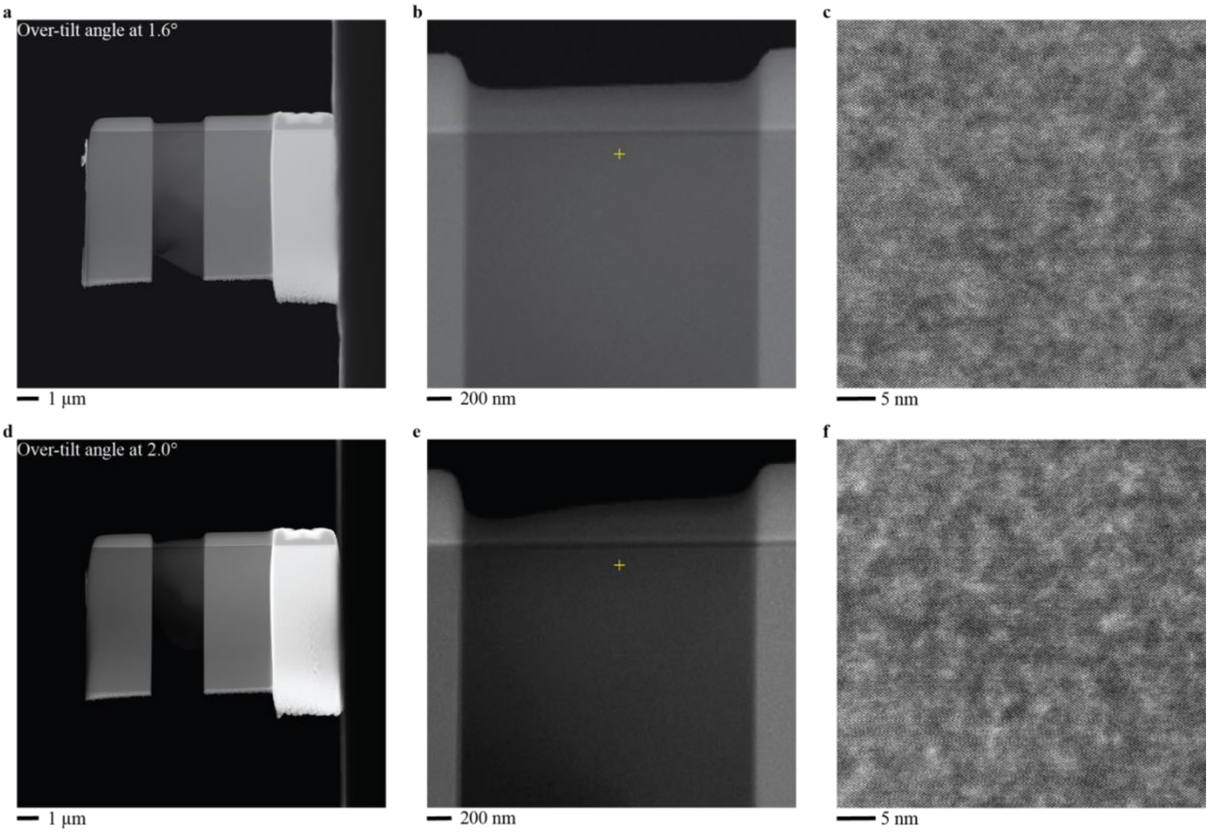

Supplementary Fig. 2 STEM results of the Si lamellae. a-c STEM results from a Si lamella, in which over-tilt angle at 2 kV-FIB is set as 1.6°. **a** A low-magnification HAADF-STEM image. **b** A zoom image of the low-magnification STEM. **c** An atomic-level HAADF image was acquired in a region near the top protective layer (+ mark in **b**) along the [110] direction. **d-f** STEM results from another Si lamella, in which over-tilt angle at 2 kV-FIB is set as 2.0°. All images were acquired by a conventional TEM, operated at 200 kV.

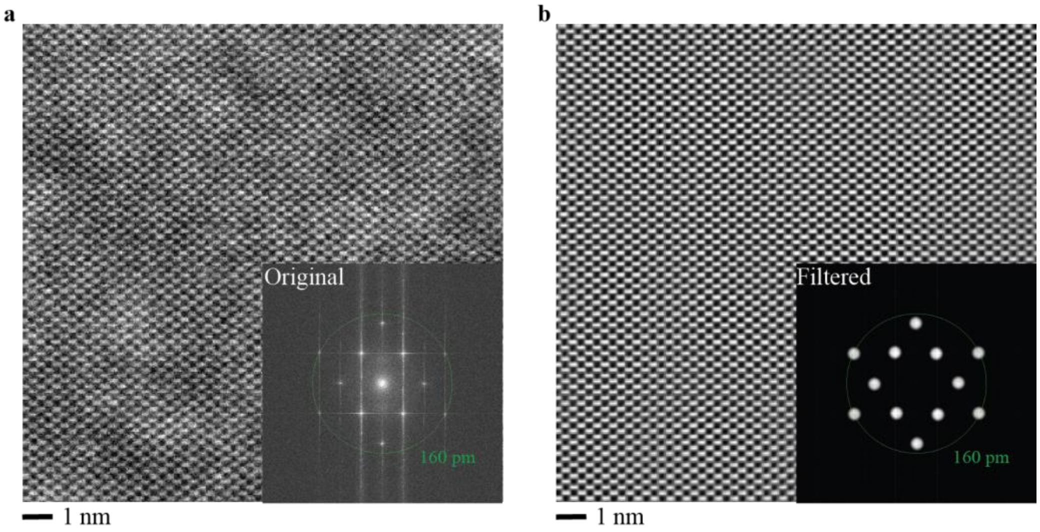

Supplementary Fig. 3 Image processing for computing score of a crystalline image. a An atomic-level STEM image of a Si lamella cropped from Supplementary Fig. 2c. Inset is the original FFT pattern of the original HAADF image. **b** A crystalline image from **a**. Original FFT pattern is masked to pass only periodic pattern of Si lattice. Inset shows the masked crystalline FFT pattern. Then, the crystalline image is computed by invert-FFT from the crystalline FFT pattern. To improve statistics, we applied the image processing to the raw STEM image with the size of 47.7 nm by 47.7 nm.

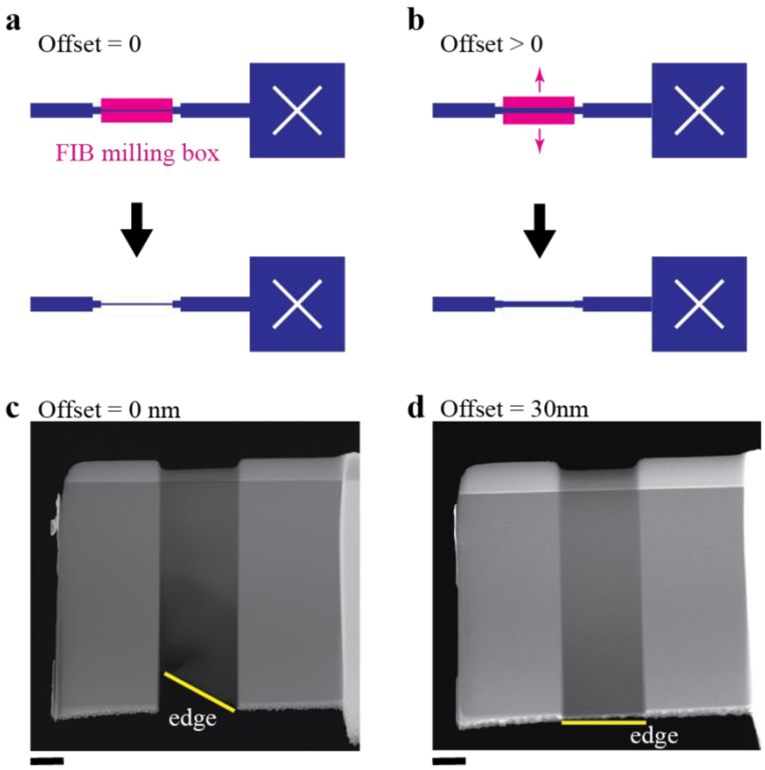

Supplementary Fig. 4 Fine-tuning of the thickness of a Si lamella by milling offset. a Schematics of top-down FIB showing the effect of changing the milling offset (both sides) in the recipe to control the sample thickness. The automation software sets the position of the FIB milling box based on the target thickness. The milling offset shifts the final FIB milling boxes away from the sample center. **b** When an offset is added (offset > 0), the resulting final thickness increases. **c, d** HAADF-STEM images of Si samples prepared using a fixed 1.6° over-tilt angle and using a 2 kV-FIB milling offset of 0 nm (in **c**) and 30 nm (in **d**). In the case of zero offset, the Si sample was over-polished and the target area was lost (see the bottom shape in **c**). With an offset of 30 nm, the bottom edge of the sample is very thin but is still present (see the bottom edge in **d**). Hence, we tune the thickness of a Si lamella by applying the milling offset of 30 nm at the final 2 kV-FIB process (where the over-tilt angle is set at 1.6°). Scale bars are 1 μm.

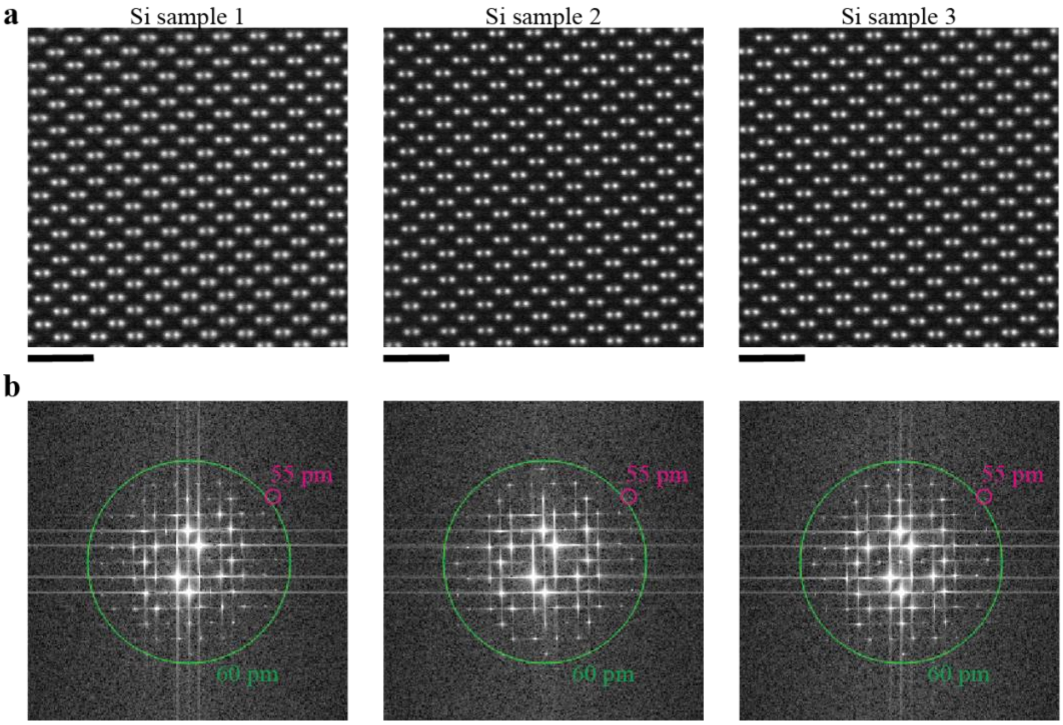

Supplementary Fig. 5 Aberration-corrected STEM imaging of Si lamellae near the top protective layer. a Atomic-level HAADF-STEM images of Si lamellae along the [110] direction. The three lamellae are the same as in Figs. 4 and 5. Each atomic-level HAADF-STEM image was acquired in regions near the top protective layer. **b** FFT patterns of **a**. Magenta circles are eye-guides of diffraction spots of 55 pm resolution. Green circles correspond to 60 pm resolution. The scale bar is 1 nm.

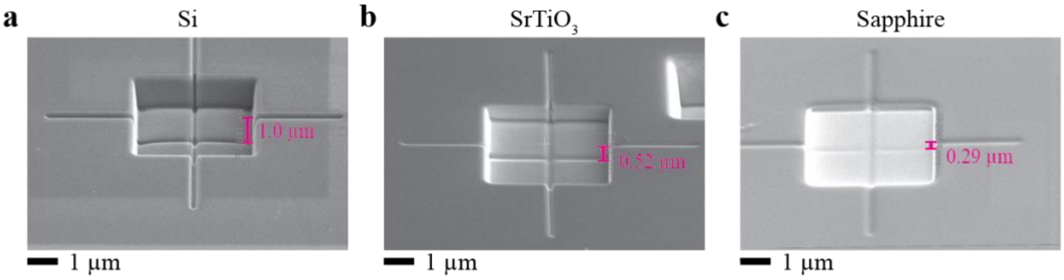

Supplementary Fig. 6 Comparing milling rates between Si, $SrTiO_3$, and sapphire. FIB at 30 kV milled bulks of Si, $SrTiO_3$, and sapphire, where FIB current and milling duration are all the same. Then, SEM measures the milling depths of Si, $SrTiO_3$, and sapphire as 1.0 μm, 0.52 μm, and 0.29 μm, respectively.
